# Bioimaging Nucleic-Acid Aptamers with Different Specificities in Human Glioblastoma Tissues Highlights Tumoral Heterogeneity

**DOI:** 10.3390/pharmaceutics14101980

**Published:** 2022-09-20

**Authors:** Elisabete Cruz Da Silva, Sophie Foppolo, Benoît Lhermitte, Marina Ingremeau, Hélène Justiniano, Lorraine Klein, Marie-Pierre Chenard, Romain Vauchelles, Basma Abdallah, Maxime Lehmann, Nelly Etienne-Selloum, Monique Dontenwill, Laurence Choulier

**Affiliations:** 1 Laboratory of Bioimaging and Pathologies, University of Strasbourg, UMR 7021 CNRS, 67400 Illkirch, France; 2 Department of Pathology, Strasbourg University Hospital, 67200 Strasbourg, France; 3 Centre de Ressources Biologiques des Hôpitaux Universitaires de Strasbourg, 67200 Strasbourg, France; 4 Laboratory of Biotechnology and Cell Signaling, University of Strasbourg, UMR 7242 CNRS, 67400 Illkirch, France; 5 EFS Grand Est, INSERM UMRS 1255, 67000 Strasbourg, France; 6 Department of Pharmacy, Institut de Cancérologie Strasbourg Europe, 67200 Strasbourg, France

**Keywords:** nucleic-acid aptamers, histofluorescence, multiplexing, cell-surface receptors, detection, EGFR, integrin α5β1, glioblastoma

## Abstract

Nucleic-acid aptamers are of strong interest for diagnosis and therapy. Compared with antibodies, they are smaller, stable upon variations in temperature, easy to modify, and have higher tissue-penetration abilities. However, they have been little described as detection probes in histology studies of human tissue sections. In this study, we performed fluorescence imaging with two aptamers targeting cell-surface receptors EGFR and integrin α5β1, both involved in the aggressiveness of glioblastoma. The aptamers’ cell-binding specificities were confirmed using confocal imaging. The affinities of aptamers for glioblastoma cells expressing these receptors were in the 100–300 nM range. The two aptamers were then used to detect EGFR and integrin α5β1 in human glioblastoma tissues and compared with antibody labeling. Our aptafluorescence assays proved to be able to very easily reveal, in a one-step process, not only inter-tumoral glioblastoma heterogeneity (differences observed at the population level) but also intra-tumoral heterogeneity (differences among cells within individual tumors) when aptamers with different specificities were used simultaneously in multiplexing labeling experiments. The discussion also addresses the strengths and limitations of nucleic-acid aptamers for biomarker detection in histology.

## 1. Introduction

Conventional immunohistochemistry (IHC) is a standard diagnostic process in tissue pathology that complements hematoxylin–eosin staining and is commonly used for tumor diagnosis, guiding patient stratification and treatment decision. This tissue-based technique is, however, limited by the labeling of only one biomarker per section of tissue. Yet, unique marker characterization is slowly becoming replaced by tumoral molecular signatures based on mRNA and protein expression data. Multiplex tissue imaging allows the detection of multiple biomarkers in the same tissue section to be performed, revealing the spatial relationships among the cells expressing these biomarkers. Various antibody-based approaches have been developed to detect together several antigens in tissue samples [1,2,3]. The most common methods use sequential colorimetric or fluorescent staining. Briefly, the classical IHC approach relies on the use of a primary antibody to detect the target of interest and an anti-species secondary antibody labeled with an enzyme or a fluorophore for signal detection. For an example of immunofluorescent detection, horse-radish peroxidase can be used to catalyze a reaction between tyramide and tyrosine residues on or near the epitope and to covalently deposit a fluorophore on the tissue section [3,4,5]. Multiplex tissue imaging can also be achieved via sequential staining rounds after the chemical or heat-mediated striping of antibodies [6]. This detection method does not require labeled primary antibodies, and IHC clinically validated antibodies can be used. Although good results have been achieved using this approach, it is time consuming; the striping rounds can damage tissues; and secondary antibodies should be carefully considered to avoid cross-reactivity. Staining protocols are simplified and performed faster using primary antibodies directly conjugated to fluorophores or metal isotopes, enzymes, oligonucleotides, etc., which can be used for detection [1]. Conjugation, however, is not a turnkey process and might lead to batch-to-batch variations.

Another approach, based on nucleic-acid aptamers, is worth exploring to detect one or different molecular biomarkers at the same time in a single tissue section. Aptamers are small RNA or ssDNA sequences that acquire a three-dimensional structure to bind to their targets with high affinities and specificities. They are also referred to as ‘chemical antibodies’ [7]. Besides their relevance for therapeutic applications [8], aptamers offer a promising field of investigation for diagnostic studies, such as histochemistry, in vivo molecular imaging, the isolation and detection of cancer cells (including circulating tumoral cells), and the identification of cellular biomarkers or circulating biomarkers in liquid biopsies [9,10,11]. Aptamers are chemically synthetized. As such, compared with antibodies, they are faster and cheaper to produce and easier to directly conjugate to a wide range of tags with high batch fidelity. Approximately 5–10 times smaller than monoclonal antibodies, they have better tissue-penetration abilities [12], which may be an advantage in histology, when the accessibility of the epitope is reduced, such as in fixed tissue [13], or for multiplexing, when steric hindrance might compromise ligand accessibility. Aptamers are thus emerging diagnostic tools to complement their protein alter egos. Despite all their advantages and since their potential for patient tissue staining was first validated in 2010 [14], only few aptamers have been described so far for staining histological tissue sections ([15] and reviewed by [13,16,17]). Moreover, to our knowledge, only one study refers to aptamer-based multiplexing in tumoral tissue [18].

We are interested in the aptamer-based fluorescent detection of glioblastoma (GBM) biomarkers. GBM is the most common and aggressive brain tumor in adults, with a median overall survival under 20 months [19]. The standard treatment, which consists of maximal tumor resection with adjuvant concomitant radio-chemotherapy, has remained unchanged since 2005 [20]. Many molecular targets have been identified, and a number of targeted therapies under clinical evaluation have been reported. However, so far, they remain inefficient [21]. GBMs, as the name suggests, are characterized by high heterogeneity. Histological features that characterize GBM are the presence of atypia, mitotic activity, increased cell density, necrosis, and the abnormal growth of blood vessels around the tumor [22]. Since 2016, the GBM diagnostic has been based on both histological and molecular characteristics according to the World Health Organization (WHO) classification of tumors of the central nervous system [23]. The recent 2021 WHO classification has added even more molecular features, and one of them to be noted in relation to our study is the EGFR amplification [24]. However, additional reliable biomarkers are urgently needed to better assess the prognosis of GBM patients, some of them being cell-surface protein biomarkers, the expression of which is often remodeled [21,25,26]. In this study, we addressed EGFR (epidermal growth factor receptor) and the α5β1 integrin.

EGFR, a 170 kDa member of the HER gene family of proteins that contains four receptor tyrosine kinases (RTKs), drives the development of solid tumors [27]. Its overexpression leads to aberrant signaling pathways promoting tumor-cell proliferation, growth, survival, differentiation, and angiogenesis. In GBM, EGFR is amplified and/or mutated in more than 40% of cases [28]. After those targeting VEGF (vascular endothelial growth factor) and VEGFR (VEGF receptor), the most frequently reported drugs in GBM targeted therapies are those targeting EGFR. Forty clinical trials in phases II–IV reported in the last 20 years were based on tyrosine kinase inhibitors and monoclonal antibodies [21,29]. Integrins, a family of αβ heterodimeric transmembrane cell-surface adhesion and signaling receptors, are implicated in cell–cell and cell–matrix communication and are expressed in all nucleated cells of multi-cellular animals [30]. In vertebrates, integrins synergize with other receptors, including RTKs. Frequently overexpressed in solid tumors, integrins promote cell survival, proliferation, invasion, and stemness maintenance and are major actors in disease progression and resistance to therapies [31,32,33,34,35]. In GBM, several integrins are overexpressed in tumoral and endothelial cells [36]. Higher expression levels of the fibronectin receptor, integrin α5β1, are observed in GBM tissue compared with adjacent normal brain tissue [37]. This overexpression was associated with GBM aggressiveness at the RNA [38,39,40] and protein levels [41].

EGFR and integrin α5β1 are two cell-surface receptors that share common features in their signaling pathways, leading to the development of compensatory mechanisms implicated in resistance to therapies targeting RTKs [32]. They are targets of therapeutic interest in the fight against the emergence of resistance. Inhibiting these receptors individually displayed poor results in GBM clinical trials [21].

**Figure 1:**
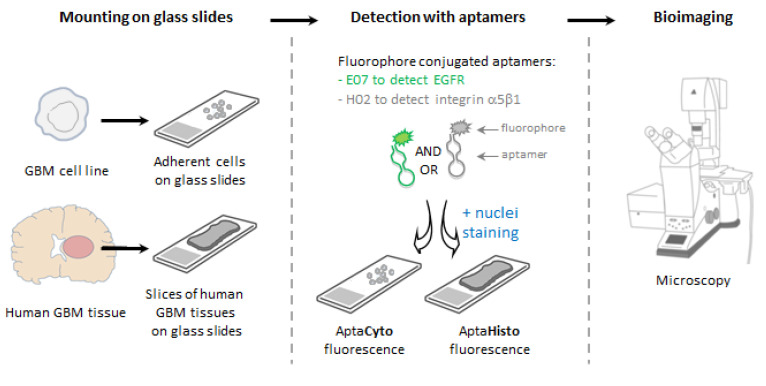
Experimental scheme illustrating the aptafluorescence experiments. After mounting GBM cells or tissues on glass, cells or tissues were incubated with aptamers covalently conjugated to fluorophores. Two aptamers with different specificities were used in this study: aptamer E07 to detect EGFR and aptamer H02 to detect integrin α5β1. At the end of this manuscript, we also describe a technique in which both aptamers were simultaneously incubated on GBM tissues (multiplexing experiments). Fluorescence microscopy was then realized for bioimaging. Drawings are not to scale.

However, combined targeted therapies would certainly prove to be more effective for this highly heterogeneous tumor [42], which emphasizes the importance of patient selection for personalized treatments. Molecular imaging techniques are needed for detecting GBM biomarkers. Our study focused on the use of fluorophore-conjugated nucleic-acid aptamers targeting EGFR and the α5β1 integrin as detection tools on GBM cells and tissues. Target expression and aptamer binding were first validated in cell lines using flow cytometry and confocal imaging. Aptamers were then further compared to antibodies and used in mono- or multiplexing experiments on formalin-fixed and paraffin-embedded human brain tissues to highlight tumoral heterogeneity. Figure 1 illustrates the experimental design of our study.

## 2. Materials and Methods

### 2.1. Materials

All nucleic-acid aptamers and chemicals were purchased from IBA Lifesciences (Goettingen, Germany), Eurogentec (Seraing, Belgium), and Sigma-Aldrich (Hamburg, Germany). The sequences of all aptamers from this study are described in Supplementary Table S1.

### 2.2. Cell Culture

Cell culture media and reagents were from Lonza (Basel, Switzerland) or Gibco (Thermo Fisher Scientific, Waltham, MA, USA). Human GBM cell line U87MG EGFR WT was kindly provided by Dr. Frank Furnari [43]. LN319, MCF7, and MDA-MB-231 were kindly provided by Pr. Monika Hegi (Lausanne, Switzerland) and Dr. Catherine Tomasetto (IGBMC, Illkirch, France), respectively. The cell lines from GBM were maintained in Eagle’s minimum essential medium (EMEM) with 10% fetal bovine serum (FBS), 1% sodium pyruvate, and 1% non-essential amino acids, in a 37 °C humidified incubator with 5% CO_2_. The MCF7 cell line was maintained in Dulbecco’s modified Eagle’s minimum essential medium (DMEM), containing 1 g/L glucose and supplemented with 10% FBS, 40 µg/mL gentamicin, and 0.6 µg/mL insulin. The MDA-MB-231 cell line was maintained in Roswell Park Memorial Institute (RPMI) 1640 medium supplemented with 10% FBS and 40 µg/mL gentamicin.

### 2.3. Western Blot

Cells were lysed in 1% Triton X-100, NaF [100 mmol/L], NaPPi [10 mmol/L], and Na_3_VO_4_ [1 mmol/L] in PBS, supplemented with complete anti-protease cocktail (Roche, Basel, Switzerland). A total of 10 µg of protein was separated on precast gradient 4–20% SDS-PAGE gels (Bio-Rad, Hercules, CA, USA) and transferred to polyvinylidene fluoride (PVDF) membranes (Amersham Bioscience, Buckinghamshire, UK). After blocking, membranes were probed with primary antibodies targeting EGFR (D38B1, #4267; Cell Signaling Technology, Danvers, MA, USA), α5 integrin (D7B7G, #98204S; Cell Signaling Technology), and glyceraldehyde 3-phosphate dehydrogenase (GAPDH; Millipore, Molsheim, France). Immunological complexes were revealed with horseradish peroxidase (HRP)-conjugated secondary antibodies (Promega, Madison, WI, USA) at a 1/10,000 dilution. Revelation was performed with enhanced chemiluminescence (ECL; BioRad) using an LAS4000 imager (GE Healthcare, Dornstadt, Germany). GAPDH was used as housekeeping protein to serve as the loading control for all cell lysate samples. The quantification of non-saturated images was performed with ImageJ software. Analyses were performed on at least three independent experiments.

### 2.4. Flow Cytometry

For the determination of equilibrium binding affinities using flow cytometry, aptamer E07 was used at different concentrations (5000, 4000, 2000, 1000, 500, 250, 100, 10, and 1 nM). After detachment with 0.2 M EDTA, 300,000 cells were incubated for 30 min with Cy5-labeled aptamers under gentle agitation to avoid cell sedimentation. Cells used as controls were incubated with Cetuximab at 1 µg/mL for 3 min, washed, and then analyzed (counting 10,000 events) using an FACSCalibur flow cytometer (Beckson Dickinson, Le Pont de Claix, France). Flowing software (version 2.5.1, Turku Bioscience, Turku, Finland) was used to analyze data. To determine the equilibrium constant, K_D_, experiments were repeated three times, and GraphPad Prism software (version 5.04, Dotmatics, San Diego, CA, USA) was used.

### 2.5. Fluorescence-Based Assays on Cell Lines

Adherent cells were plated on sterile glass coverslips for one night at 37 °C in culture medium, washed three times, and then saturated for 1 h at room temperature (RT) in selection buffer (phosphate-buffered saline, 1 mM MgCl_2_, 0.5 mM CaCl_2_; pH 7.4) containing 2% BSA. Labeled aptamers were denatured at 95 °C for 3 min, incubated on ice for 5 min before being resuspended in selection buffer, and applied to cells for 30 min at 37 °C. Cells were then washed in selection buffer, fixed for 8 min in 4% paraformaldehyde (PFA), permeabilized for 2 min with 0.2% Triton, and washed again. Then, immunocytochemistry was performed with the following primary antibodies: anti-EGFR (clone D1D4J; Cell Signaling Technology; 1/200) and anti-EEA1 (early endosome antigen 1; clone 14/EEA1; BD Transduction Laboratories; 1/1000). Primary antibodies were added overnight (O/N) at 4 °C, followed by two washes and incubation for 1 h at RT with a secondary antibody conjugated to Alexa 488 or 568 (Life Technologies, Carlsbad, CA, USA) at a 1 µg/mL final concentration. DAPI was added at 1 µg/mL to visualize nuclei. Washing steps were performed before mounting using fluorescent mounting medium (S3023; Dako, Carpinteria, CA, USA).

### 2.6. Human Tissue Samples

Twenty patients’ histologic fresh-frozen, formalin-fixed, paraffin-embedded GBM tissues were obtained from the tumor collection of the pathology department of Strasbourg University Hospital (Centre de Ressources Biologiques des Hôpitaux Universitaires de Strasbourg; declaration number DC-2016-2677t) after obtaining written informed consents from patients. Twenty hematoxylin–eosin-stained paraffin-embedded human tissues, examined by one neuropathologist (B.L.), were confirmed as GBMs according to the 2021 WHO classification of tumors of the central nervous system [24]. Two human epileptic brain tissue samples were used as non-tumoral tissues. Negative controls were performed either with DAPI alone or, for immunolabeling experiments, without adding primary antibodies (i.e., only secondary antibodies were added).

### 2.7. Fluorescence-Based Labeling Assays on Human Tissue Samples

Apta- and immunostaining were realized using tissue sections mounted on glass slides. Paraffin-embedded sections were deparaffinized, rehydrated through a graded alcohol series, and subjected to an antigen unmasking protocol. Briefly, sections were boiled at 100 °C for 10 min in target retrieval solution at pH 9 (S2367; Dako), cooled down to RT for 20–40 min, and rinsed briefly in H_2_O; then, they were washed in selection buffer. Fresh-frozen sections were fixed in 4% PFA for 10 min at RT and then washed in selection buffer. For aptafluorescence, slides were rinsed for 5 min in H_2_O and then in blocking buffer (selection buffer, 2% BSA) in the presence or not of 100 µg/mL tRNA from baker’s yeast (R56-36; Sigma-Aldrich, Hamburg, Germany) or yeast tRNA plus salmon sperm DNA (D1626; Sigma-Aldrich) for 1 h in a humid chamber at RT; they were rinsed in H_2_O, followed by selection buffer, and drained. Aptamers were denatured at 95 °C for 3 min and incubated on ice for 5 min before dilution in selection buffer to a final concentration of 1 or 2 µM for aptamer H02 targeting the α5 integrin and 500 nM for aptamer E07 targeting EGFR. Aptamers were incubated in tumor sections for 1 h on ice, briefly washed in selection buffer, drained, fixed in 4% PFA, and then washed three times in PBS. For immunofluorescence, slides were rinsed briefly in PBS, washed for 5 min in PBS-T (0.1% Tween-20 in PBS), drained, and then incubated in blocking buffer BB-I (5% goat serum in PBS, 0.1% Triton X-100) for 1 h in a humid chamber. O/N incubation with anti-integrin α5 mAb 1928 (6B8516; Millipore, Molsheim, France; 1/200) in BB-I was followed by 3 washes of 3 min in PBS-T and by an incubation step with a 1/500 dilution of a secondary antibody raised against the host species used to generate the primary antibodies, conjugated to Alexa Fluor 488 or 647 (ThermoFisher Scientific, Braunsweig, Germany; A-21245, A-11008, or A-11004) in BB-I. Immuno- and aptastaining were followed by staining with DAPI at a 1 µg/mL final concentration for 30 min at RT to visualize cell nuclei. Stained samples were then washed in PBS. Coverslips were mounted using fluorescent mounting medium (S3023; Dako).

### 2.8. EGFR Immunostaining of Human Tissue Samples

EGFR immunostaining was performed on deparaffinized GBM sections with BenchMark Ultra (Ventana, Roche, Basel, Switzerland). After pre-treatment with Protease 1 for 8 min, the monoclonal antibody clone E30 (DAKO), reactive against the extracellular domain of the EGFR protein, was used at a dilution 1/500 for 32 min. The detection ultraview DAB system was used for revelation. Negative controls omitting the primary antibody were included.

### 2.9. Imaging

Images of apta- and immunofluorescence were acquired using a NanoZoomer S60 digital slide scanner (Hamamatsu Photonics, Iwaka, Japan) and/or a Leica TCS SPE II confocal microscope at 20× or 63× (oil immersion) magnification. For all slide scanning, images were processed at different magnifications using NPD.view2 version 2.7.43. Mean integrated fluorescence intensity on cells and tissues was measured using ImageJ software as previously described [41,44]. The plot profile tool in ImageJ (version 1.50f, U.S. National Institutes of Health, Bethesda, MD, USA) was used to display a 2D histogram of the intensities of pixels along a line drawn within an image. The statistical analysis of data was performed with ANOVA. Data were analyzed with GraphPad Prism version 5.04 and are represented as means ± SEMs. Hematoxylin–eosin tumors were read using PathScan Viewer software.

## 3. Results

### 3.1. Validation of Target Expression and Aptamer Binding to Cell Lines

We recently published the identification of aptamer H02 targeting integrin α5β1 [44]. Its affinity for GBM cell line U87MG expressing integrin α5 was determined using flow cytometry (K_D_ = 277.8 ± 51.8 nM; Table 1). Using confocal imaging, we showed that this aptamer was able to discriminate among ten GBM cell lines expressing high and low levels of integrin α5. Similarly, in the present study, we first characterized the binding parameters of aptamer E07 targeting EGFR [45] in GBM cells.

**Figure 2:**
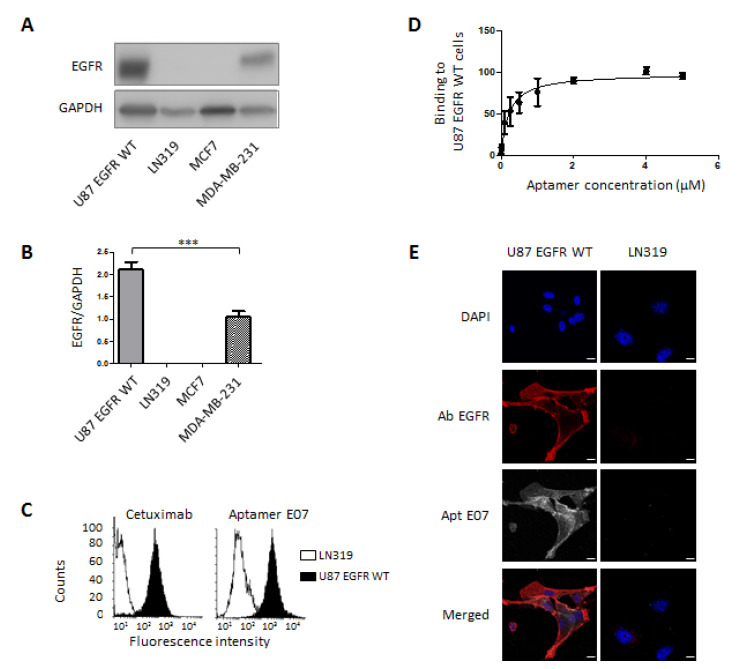
Validation of EGFR expression and E07 aptamer binding to cancer cells. (**A**,**B**) Immunodetection of EGFR in different cancer cell lines. (**A**) Immunoblot showing the expression of EGFR (175 kDa) in U87 EGFR WT, LN319, MCF7, and MDA-MB-231 cell lines. GAPDH (37 kDa) was used as loading control. (**B**) Quantitative immunoblot analysis. Histograms represent the means ± SDs of three independent experiments normalized to GAPDH, with *** *p* < 0.005 (non-significant data are not specified). (**C**,**D**) Flow cytometry experiments. (**C**) Left side: Control of EGFR expression via the binding of EGFR antibody Cetuximab conjugated to Cy5 to U87 EGFR WT (black fill) and LN319 cells (black line, white fill). Right side: Comparison of the binding profiles of aptamer E07-Cy5 at 1 µM to U87 EGFR WT cells (black fill) and LN319 cells (black line, white fill). (**D**) Titration of aptamer E07. Different concentrations of the E07-Cy5 aptamer (0.001, 0.01, 0.1, 0.25, 0.5, 1, 2, 4, and 5 µM) were incubated with a constant number of U87 EGFR WT GBM cells and analyzed using flow cytometry. Titration resulted in the determination of the equilibrium affinity parameter, K_D_, for the interaction between U87 EGFR WT cells and aptamer E07 (208.7 ± 45.57 nM). (**E**) Confocal imaging of E07-Cy5 aptamer in two cell lines, LN319 and U87 EGFR WT. Cells were seeded in coverslips and incubated with 100 nM of E07-Cy5 aptamer for 30 min (white). The incubation of antibody anti-EGFR was followed by incubation with a secondary antibody labeled with Alexa 568 (represented in red). Nuclei were stained with DAPI (blue). Scale bar = 10 μm.

Immunoblots showed that EGFR was expressed in U87 EGFR WT cells but was absent in LN319 (Figure 2A,B). EGFR detection by means of flow cytometry in both cell lines was controlled using anti-EGFR antibody Cetuximab conjugated to Cy5 (Figure 2C, left). The shift in fluorescence intensity to the left confirms the low expression level of EGFR in LN319 compared with the U87 EGFR WT cell line. This difference in fluorescence intensity was also observed for the binding of Cy5-conjugated aptamer E07, named E07-Cy5 (Figure 2C, right). The equilibrium affinity parameter, K_D_, of the interaction between E07-Cy5 and U87 EGFR WT cells was determined using flow cytometry (Figure 2D). Briefly, binding events associated with the fluorescence signal of different concentrations of aptamers, ranging from 1 nM to 5 µM, to a constant number of cells were measured. A K_D_ of 208.7 ± 45.6 nM was determined by plotting the mean fluorescence of U87 EGFR WT cells against the concentration of the E07 aptamer (Figure 2D, Table 1). For confocal assays, confluent cells were stained with E07-Cy5 at 100 nM for 30 min. After cell fixation, cells were immunolabeled with an anti-EGFR primary antibody and then with a secondary antibody labeled with Alexa 568. The specificity of the E07-Cy5 aptamer was characterized on the two GBM cell lines, U87 EGFR WT and LN319, expressing high and low levels of EGFR, respectively (Figure 2E). Confocal imaging was also performed on other cell lines: breast cancer cell lines MCF-7 and MDA-MB-231 (Figure S1). MDA-MB-231 expressed an intermediate level of EGFR, whereas EGFR was not immunodetected in MCF7 (Figure 2A,B). Confocal imaging shows that aptamer E07 detected EGFR on U87 EGFR WT (Figure 2E) and to a lesser extent on MDA-MB-231 cells (Figure S1). Clearly, EGFR aptalabeling corresponded with EGFR immunolabeling and reflected well the EGFR expression level in these cell lines. Fluorescently labeled aptamer E07 was not detected in the cell lines that did not express EGFR (LN319 and MCF7).

On the basis of their specific cell-binding properties to their respective receptors, we considered the two aptamers, H02 and E07, suitable for integrin α5β1 and EGFR detection in human GBM tissues.

### 3.2. Apta- and Immunodetection of Integrin α5β1 in Paraffin-Embedded and Frozen Glioblastoma Sections

**Figure 3:**
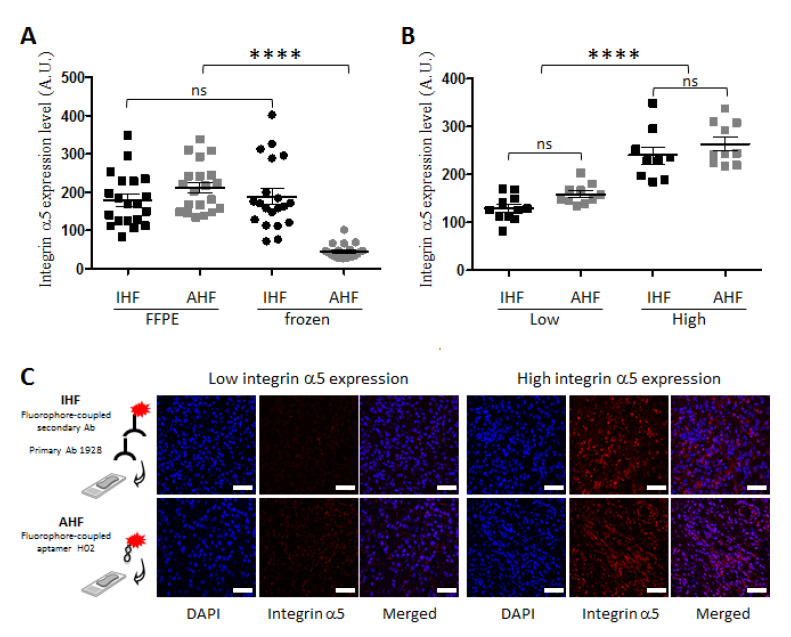
Quantification of integrin α5 expression level in 20 glioblastoma tissues using immunohistofluorescence (IHF) and aptahistofluorescence (AHF). (**A**,**B**) Distribution of cumulative data obtained via IHF (black) and AHF (gray) (**A**) of formalin-fixed paraffin-embedded (FFPE; squares) and frozen (circles) sections and (**B**) of FFPE sections only, considering samples showing high or low integrin α5 expression levels expressed as arbitrary units (A.U.). Statistical analyses were performed with Student’s *t* test (**** *p* < 0.0001; ns, not significant). (**C**) Representative images of low and high integrin α5 expression staining via IHF and AHF are represented (magnification × 40). The drawings on the left (not to scale) symbolize the detection in tumor sections using IHF (as an indirect method of detection, with Ab 1928 and a fluorophore-conjugated secondary antibody) and AHF (as a direct detection method, with fluorophore-coupled aptamer H02). Integrin α5 labeling is represented in red. Nuclei were stained with DAPI (blue). Scale bar = 50 μm.

We investigated whether the conditioning of the tumor sections had an influence on aptalabeling using 20 tumor sections from GBM patients. Formalin-fixed paraffin-embedded (FFPE) sections were deparaffinized, rehydrated, and subjected to an antigen unmasking protocol. Fresh-frozen sections were fixed in 4% paraformaldehyde. Aptafluorescence and, for comparison, immunofluorescence experiments were performed to detect integrin α5β1 using the cyanine 5-conjugated H02 aptamer, named H02-Cy5, at 2 µM and anti-integrin α5 mAb 1928 followed by a secondary antibody coupled to Alexa 647. mAb 1928 was recently used to detect integrin α5 via the immunostaining of GBM-PDX and FFPE tissues [41,44]. Nuclei stained with DAPI allowed us to select several fields per tumor section with homogeneous tissue distribution for quantification. The integrin α5β1 protein expression level was quantified in each sample using the mean fluorescence intensity (MFI) as recently described using confocal imaging for aptahistofluorescence (AHF) [44] and for immunohistofluorescence (IHF) [41]. IHF showed similar results for FFPE and frozen tissue sections. Similar results were also obtained via IHF and AHF for FFPE sections (Figure 3A). These results highlight a good reproducibility of IHF regardless of tumor section conditioning. They also emphasize the ability of aptamer H02 to detect integrin α5β1 in human FFPE GBM sections. However, the AHF intensities of frozen sections were too low for the detection of integrin α5β1 with aptamer H02 and to be compared with data on FFPE sections (Figure 3A). In the subsequent phases of this study, only FFPE sections were further studied.

### 3.3. Detection of Integrin α5β1 Using Apta- and Immunohistofluorescence on FFPE GBM Sections Highlighted Inter-Tumoral Heterogeneity

A recent analysis of integrin α5 expression revealed its upregulation as a negative prognostic biomarker of GBM; the analysis was part of a study of the relationship between patient outcome and α5 protein expression levels in a cohort of 95 FFPE GBM sections using IHF [41]. To define the cut-off threshold allowing one to distinguish two groups characterized by low and high integrin α5 expression levels, the median of the MFI (MMFI) was used. In this present study, the same method was applied to compare AHF and IHF on 20 FFPE GBM sections, different from [41]. The distribution of data is shown in Figure 3B, and representative images of sub-populations with IHF and AHF are shown in Figure 3C. Two groups are clearly distinguished, both via IHF and AHF. Moreover, the values of the ratio of high versus low MMFI were similar for IHF (1.8) and AHF (1.6) and matched the value of 1.5 obtained by Etienne-Selloum et al. [41]. The GBM inter-tumoral heterogeneity illustrated by these results is just as likely to be shown with antibody 1928 via IHF or aptamer H02 via AHF. These results demonstrate that imaging and quantifying inter-patient heterogeneity based on integrin α5β1 detection is similarly achievable in FFPE GBM sections, using either an antibody or an aptamer.

### 3.4. Aptahistofluorescence to Highlight Intra-Tumoral Heterogeneity

Because of it being a likely major cause of treatment resistance, we then assessed whether intra-tumoral GBM heterogeneity could be detected separately using H02 and E07 aptamers, both of them conjugated to Cyanine 5. The data obtained with aptamers were compared to immunological detection in FFPE tumor sections.

**Figure 4:**
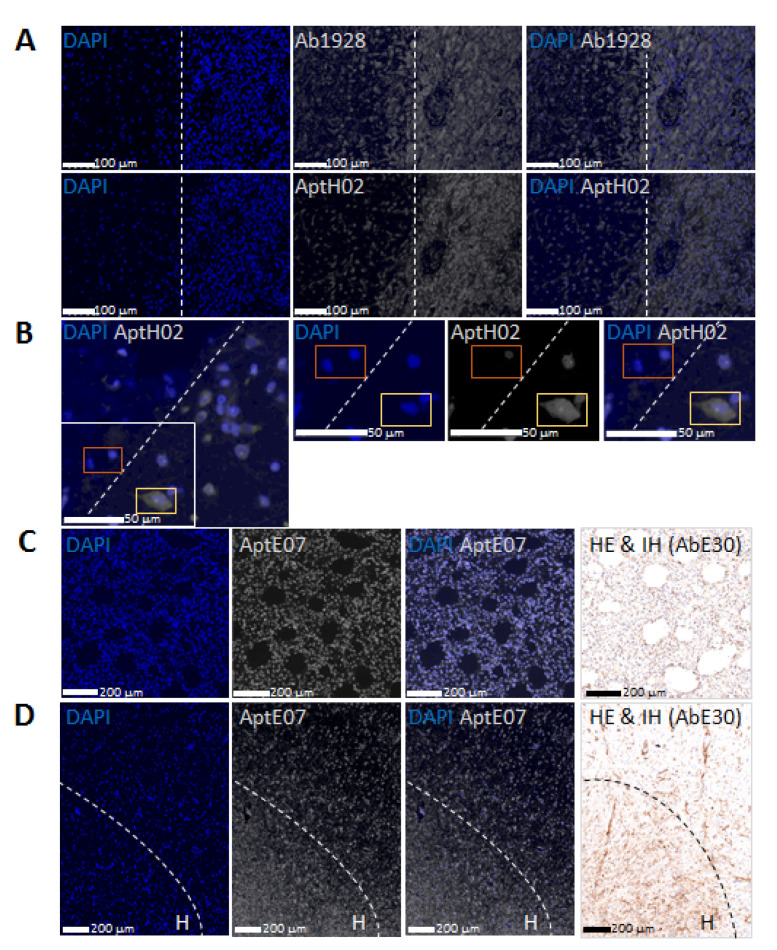
Imaging of intra-tumoral heterogeneity with aptamers targeting integrin α5 and EGFR. (**A**) Comparison of IHF and AHF for the detection of integrin α5. Equally scaled images taken with a Nanozoomer S60 slide scanner of two adjacent sections of the same tumor allowed us to perform a direct comparison between the fluorescence patterns of cells stained using IHF with antibody 1928 (Ab1928) and an Alexa647-conjugated secondary antibody and using AHF with Cyanine5-conjugated aptamer H02 (AptH02). Detection of integrin α5 is represented in white. DAPI staining is shown in blue. The dotted line delimits two areas with a small and a large number of cells on the left and right sides of the images, respectively. Another representation showing the number of cells in the two areas is provided in Figure S3. Scale bar = 100 μm. The light microscopy result of an adjacent section is shown in Figure S2. (**B**) Detection of integrin α5 using AHF. This area further shows in more detail two zones delimited by a dotted line: no or very low integrin α5 on the left side and integrin-α5 positive cells on the right side. Magnified images are from the insert, either in single-channel mode or in merged-channel mode. Integrin α5 was detected with Cyanine5-conjugated aptamer H02 (AptH02), represented in white. DAPI staining is represented in blue. The orange and yellow squares show cells unlabeled and labeled with aptamer H02, respectively. Scale bar = 50 μm. (**C**,**D**) Comparison of AHF (first three images) and immunohistochemistry (image on the right side) for the detection of EGFR. The same zone of the same tumor, identified in non-adjacent sections via fluorescence and light microscopy images, shows similar profiles for EGFR aptamer and antibody staining. Detection was realized using AHF with Cyanine5-conjugated aptamer E07 (AptE07; in white), and nuclei were stained with DAPI (in blue) and using immunohistochemistry with antibody E30 (AbE30) and a horseradish-peroxidase-conjugated secondary antibody. Scale bar = 200 μm. Images in (D) show two areas with high (noted with H) and low cell density.

Equally scaled images taken with a Nanozoomer S60 slide scanner showed a very similar staining pattern via AHF with the H02-Cy5 aptamer and via IHF with mAb 1928, followed by a secondary antibody conjugated to Alexa 647. Figure 4A shows two sections of the same tumor slice. Two areas could be identified, with a small and a larger number of cells on the left and on the right of the images, respectively, showing invading cells in the lengthwise central part. A blood vessel was visible in the right median area. As with mAb 1928, aptamer H02 allowed us to distinguish tumoral cells at the tumoral core, invading cells at the invasion border, and the edges of a blood vessel. Integrin α5β1 is indeed expressed by tumoral vessels besides its expression by GBM tumoral cells [46]. Light microscopy with H&E staining of the same area is shown in Figure S2. The comparable staining patterns using IHF and AHF further supported the specificity of aptamer H02 labeling. Furthermore, the representative image in Figure 4B shows mosaic protein expression, with cells detected by aptamer H02 and with others that were not. These AHF experiments, therefore, enabled the detection of α5+ and α5− cells within the same tumor sections, which, to our knowledge, had never been imaged.

We also compared EGFR apta- and immunodetection with the E07-Cy5 aptamer or with antibody clone E30 and a horseradish-peroxidase-conjugated secondary antibody. The anti-EGFR antibody and methodology were those used in clinics for EGFR in vitro diagnostic. As far as we know, aptamer E07 has never been reported to detect EGFR in ex vivo experiments. Both the E07 aptamer and the E30 antibody are known to detect the extracellular domain of EGFR proteins [45,47,48]. Corresponding areas from the same tumor showed similar profiles for EGFR aptamer and antibody staining using fluorescence and light microscopy of the tumoral core (Figure 4C) and invasive border (Figure 4D).

The detection profiles of integrin α5β1 and EGFR were similar using aptamers and antibodies and revealed that the expression of these two proteins was not homogeneous within tumor sections. The two aptamers used in this study were as effective as specific antibodies in demonstrating the heterogeneous staining pattern within the tumor. We, thus, validated the use of aptamers in aptafluorescence for the detection of two molecular biomarkers and to highlight tumoral heterogeneity in FFPE GBM sections.

### 3.5. Multiplexing with Aptamers with Different Specificities

**Figure 5:**
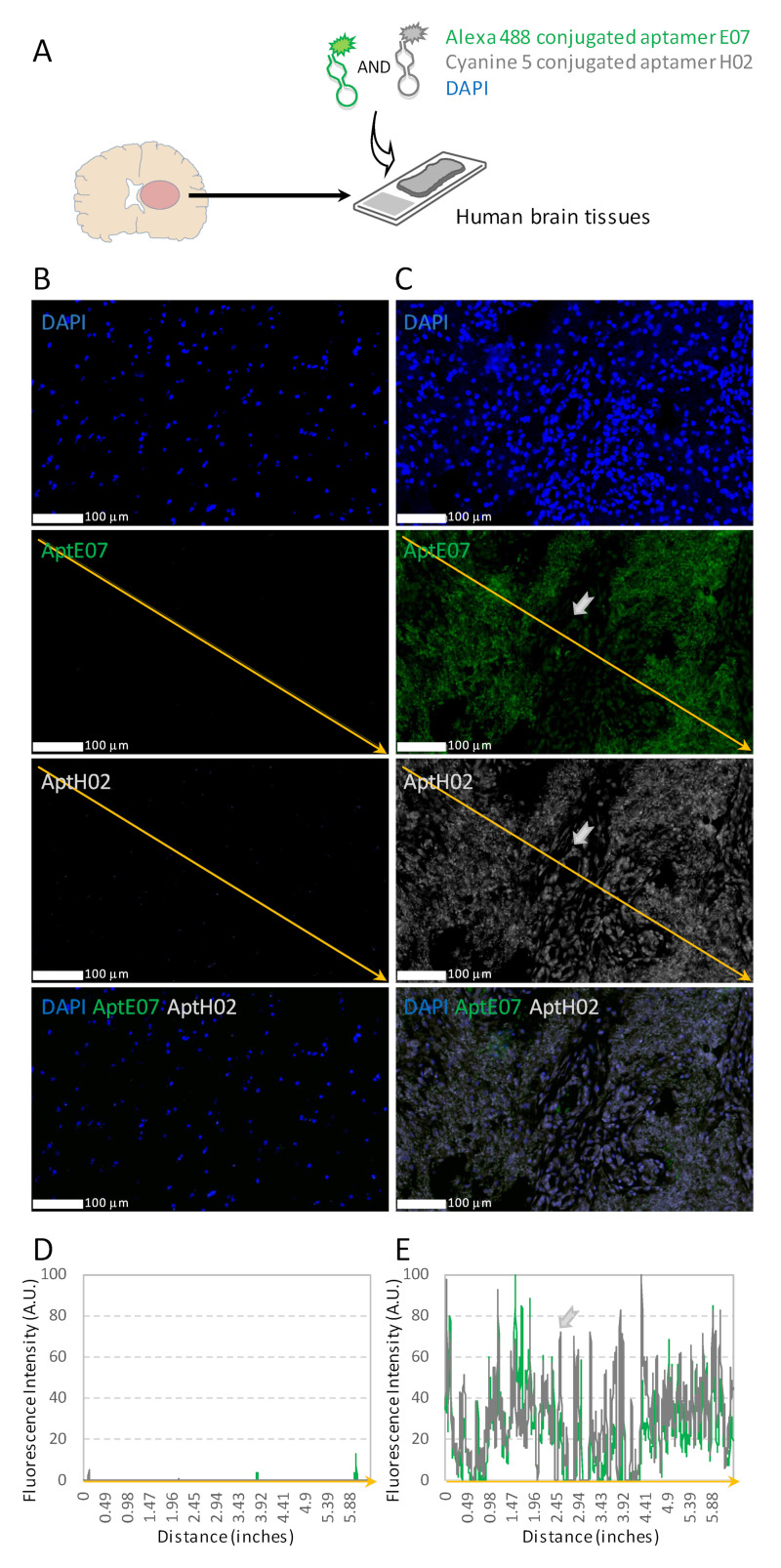
Dual labeling with aptamers targeting integrin α5 and EGFR. (**A**) Schematic depicting detection via AHF simultaneously using two aptamers, aptamers E07 and H02, conjugated to two different fluorophores (not to scale). In (**B**,**C**), we show human epileptic brain and GBM tissues, respectively. DAPI staining is shown in blue. Detection of EGFR with Alexa 488-conjugated aptamer E07 is represented in green. Detection of integrin α5 with Cyanine5-conjugated aptamer H02 is represented in gray. Images in (**B**,**C**) were captured using the same settings to allow us to perform a direct comparison of the staining intensity with a Nanozoomer S60 slide scanner. Scale bar = 100 μm. (**D**,**E**) Histograms of normalized fluorescence intensities corresponding to detection with aptamers E07 (in green) and H02 (in gray). Histograms in (**D**,**E**) correspond to the fluorescence intensities of **B** and **C**, respectively, quantified along the orange diagonal arrow. Histograms show only sparse fluorescence in epileptic tissue (**D**); they show, in GBM tissue (**E**), that areas were not uniformly labeled with both aptamers. For example, the gray arrow in (**E**) shows an area strongly and faintly labeled with aptamers H02 and E07, respectively. This area corresponds to the cells pointed at by the gray arrow in (**C**).

Since we demonstrated that aptamers H02 and E07 were separately able to detect integrin α5β1 and EGFR, we proposed their simultaneous use in the same tissue sections. In these multiplexing experiments, aptamer H02 was conjugated to cyanine 5 and aptamer E07 to Alexa 488 (Figure 5A). To avoid potential hybridization between them, aptamers H02 and E07 were heat-denatured at 95 °C and renatured separately; then, they were pooled shortly before their application to tissue sections.

Representative images of epileptic brain and GBM tissues are shown in Figure 5B,C, respectively, and the analyses of fluorescence intensities are quantified in Figure 5D,E. While E07 and H02 aptamers did not label non-tumoral tissues (Figure 5B,D), they were efficient in detecting cells expressing EGFR and integrin α5β1 within the tumor. Figure 5C,E are of particular interest. Two different patterns were observed. (i) In most areas, all cells were labeled with the two aptamers. This result highlighted, using bioimaging, the already known co-expression and potential crosstalk between EGFR and integrin α5β1 in GBM [32]. (ii) However, in some areas, such as the one shown with the gray arrow in Figure 5C,E, one could note a lower fluorescence intensity obtained with the E07 aptamer than in the side areas, which highlighted that dual apta-labeling was not identical among cells within the tumor. This indicated a differentiated expression of both receptors, i.e., equal levels of integrin α5β1 but lower levels for EGFR in this zone compared with adjacent areas.

Hence, these results showed not only areas of co-expression of EGFR and integrin α5β1 but also areas where one of these two biomarkers was underexpressed compared with the other, and this was made possible in patient tumor sections using multiplex aptamer detection.

## 4. Discussion

Tumoral heterogeneity, which encompasses both inter-tumoral heterogeneity (differences observed at the population level) and intra-tumoral heterogeneity (differences among cells within individual tumors), affects treatment response. It is the key to understand treatment failure, notably in GBM, where multiple distinct populations of tumoral cells confer survival advantage as well as resistance to therapies and for which drug treatment remains largely inefficient. Technical advances have helped to reveal GBM heterogeneity at the DNA and RNA levels. However, as gene expression data do not often highly correlate with variations in protein expression, reliable and easily implementable methods are needed to identify molecular targets at the protein level [49]. A large amount of information is missing in histology due to methodological and tool limitations. Though essential for a better understanding of pathological processes and for the development of personalized therapeutic strategies, the simultaneous detection of multiple biomarkers is not systematically studied [50]. The detection of multiple proteins in IHC, the standard method for the in situ detection of FFPE tissue, is performed on consecutive sections. The localization of different biomarkers is particularly difficult when sections are not successive, and the co-localization of markers cannot be assessed at the level of the single cell [3]. Moreover, antibodies, used for the last 40 years, have been proven to be at times unreliable, mainly due to reagent variations [9]. High-quality, reliable molecules are essential for detection, and a transition towards affinity molecules defined by their sequence has recently been proposed [51,52]. For histofluorescence multiplexing approaches, aptamers appear to be particularly suitable. Due to their smaller size compared with antibodies, they can better penetrate in tissues [12]. Aptamers are chemically synthetized, which means that they do not vary from batch to batch. Fluorophores can easily be directly conjugated to aptamers, and these constructs are detected in multiplexing fluorescent experiments when aptamers with different specificities are conjugated to different fluorophores. The AHF technique is fast and easy to implement, and our results highlight its use to detect GBM heterogeneity in FFPE tissue samples. However, a number of considerations must be taken into account to avoid the misinterpretation of the histological data.

A very recent comparative analysis of cell-surface-targeting aptamers indicated that the characterization of many of these molecules was largely confounded by a lack of uniform assessment. Kelly et al. [53] compared the ability of 15 different aptamers from the literature and surveyed them particularly for their in vitro cell-binding capacities. The targets included PSMA, EGFR, hTfR, HER2, AXL, EpCAM, and PTK7. Only 5 out of the 15 aptamers showed receptor-specific activity, and among these five aptamers was aptamer E07, which supported the selection of this aptamer in our experiments. As in this study, we considered the use of well-documented aptamers to be important, particularly those studied for their binding to identified biomarkers on cells, to have a better chance to find them to be suitable for histological detection. Aptamers are identified through an in vitro evolution process called SELEX, which stands for ‘Systematic Evolution of Ligands by EXponential Enrichment’ [54,55]. It starts with an initial RNA or ssDNA library containing 10^14^–10^15^ oligonucleotides and involves iterative cycles of selection towards targets, including small molecules, proteins, peptides, toxins, whole cells, and tissues. Different SELEX processes have been developed for the selection of aptamers targeting tumor biomarkers, with the two main ones being protein- and cell-SELEX [56]. Another selection method allows one to identify aptamers on tissues, called tissue-SELEX. This method is the best suited for further applications of selected aptamers in histology. However, the a posteriori identification of molecular targets has rarely been performed [18,57] and is difficult to achieve. In our study, we, therefore, chose aptamers already well characterized in the literature for their cell-binding properties, namely, aptamers E07 and H02. Moreover, upstream of histofluorescence, we supplemented published data with cytofluorescence experiments using flow cytometry and confocal imaging. We used appropriate receptor-expressing GBM cells and included negative cells for receptor expression (Figure 2). The affinities of aptamers for their targets were determined under conditions that were as close as possible to ‘natural’ conditions (i.e., affinities for cells). We showed that K_D_ of aptamer H02 differed 3.8-fold in the interactions aptamer–recombinant integrin α5β1 and aptamer–cell [44]. This difference was much higher for aptamer E07, as a very high binding affinity (2.4 ± 0.2 nM) was determined for the interaction between [α-32P]-ATP-labeled aptamer E07 and the recombinant human EGFR protein using filter binding assays [45], while much lower affinities were determined for the interaction between aptamer E07 and the U87 EGFR WT cell line (Table 1; 208.7 ± 45.6 nM) or EGFR-expressing pancreatic cells (26–67 nM [48]). These differences may have certainly been due to the different techniques used, but they may have also been due to the differences in the conformations of soluble recombinant proteins and cell-surface proteins, to the functional bioavailability of receptors in a cellular context, and thus to the different SELEX process used for aptamer identification, i.e., hybrid-SELEX, composed of cell- and protein-SELEXs, and protein-SELEX, for the identification of aptamers H02 [44] and E07 [45], respectively. Nevertheless, the cellular affinities determined in our study were of the same order of magnitude as those reported in the literature for the interaction of most aptamers targeting cell-surface receptors [56].

Then, since aptamers, similarly to antibodies, might recognize epitopes on cells and not on FFPE tissues, immunolabeling was conducted alongside aptahistofluorescence with antibodies and aptamers with the same specificities (Figure 3 and Figure 4). An indirect method was used for immunolabeling, which consisted of the successive incubation of anti-α5 or anti-EGFR antibodies followed by secondary antibodies. AHF is a direct method, as aptamers are directly conjugated to fluorophores; it is, therefore, faster than IHC. The binding intensities determined using AHF correlated with the localization of EGFR and integrin α5β1 detected using immunolabeling. Moreover, the labeling of GBM tissues with aptamer H02 targeting integrin α5β1 confirmed the results previously obtained with anti-integrin α5β1 antibody 1928 [41], highlighting inter-patient heterogeneity. In our study, we did not observe the superior staining of a single aptamer compared with primary antibody staining, as recently described by Gomes de Castro et al. using super-resolution microscopy [58], but rather similar staining for cell receptors was detected with aptamers in comparison with antibodies using confocal imaging and a digital slide scanner. Within the same GBM section, by means of AHF using H02, we observed intra-tumoral heterogeneity, showing that different regions of the same tumor contained cells with different protein expression levels. Different areas were observed: (i) some very intensely labeled in the tumoral core and in perivascular areas and (ii) others with less labeling in the tumor periphery, where invading cells could be detected, (iii) but also areas with cells that did not express integrin α5β1.

Last but not least, the issue of autofluorescence must be considered before performing AHF and/or IHF experiments on tissues, as it complicates the data analyses. The natural fluorescence of red blood cells occurs at several wavelengths, so the distinction between test fluorescence and endogenous fluorescence is difficult [59]. Areas and at times even whole tumor sections that were highly necrotic could not be analyzed in AHF and IHF with fluorescent reporters that absorbed light at wavelengths below 600 nm. Practically, classical controls were performed; these consisted of the analysis of slices stained with DAPI alone or without the addition of primary antibodies for immunolabeling experiments and imaged with three filters. In addition, for EGFR and integrin α5β1 detection, we performed experiments with secondary antibodies and aptamers, both conjugated to Cyanine 5 or Alexa 647, as autofluorescence was absent, with far-red-emitting dyes (optical windows above 600 nm, as recommended [59]). Thus the selectiveness of the aptamers could be analyzed and compared to that of the antibodies in adjacent slices. For multiplexing experiments, to simultaneously detect integrin α5β1 and EGFR in the same slice, we used aptamer H02 conjugated to Cyanine 5 and aptamer E07 conjugated to Alexa 488, respectively. Hence, the use of the E07 aptamer conjugated to cyanine 5 or Alexa 488 allowed the data to be compared, thus invalidating areas with autofluorescence.

A few studies describe aptamers for multiplexing experiments. For example, the seminal paper by Dr. Zu and his team showed the combination of an aptamer targeting CD4 and antibodies to phenotype cells from lymph nodes, bone marrow, and pleural fluid [60]. However, to our knowledge, only one other multiplexing study simultaneously combining two or more aptamers on pathological human solid tissue has been carried out so far. Zamay and collaborators identified three DNA aptamers to post-operative lung carcinoma tissues [61], described their use in AHC for tumoral tissue characterization, and proposed that a pair of aptamers able to bind to tumor stroma be used for tumor intraoperative visualization [18]. In our study, having ensured that H02 and E07 aptamers could detect integrin α5β1 and EGFR, respectively, on cells and tissues, having compared their tissue detection efficiency to that of antibodies specific to integrin α5β1 and EGFR, and having checked their tissue binding profile when coupled to different fluorophores, we finally evaluated them in multiplexing experiments. The multi-detection experiments consisted in simultaneously labeling the two biomarkers, integrin α5β1 and EGFR, with the two aptamers, H02 and E07, covalently conjugated to two different fluorophores emitting at different and non-overlapping wavelengths (Alexa 488 for E07 and Cyanine 5 for H02). In practice, the aptamers were heated and then cooled separately to avoid inter-aptamer pairing; then, they were mixed and deposited on the GBM sections. Our results on human GBM tumoral tissues showed two different profiles: homogeneous or heterogeneous staining (Figure 5). The labeling of cells with both H02 and E07 aptamers suggested that they expressed both integrin α5β1 and EGFR. Other tumor areas showed a less uniform pattern, with one of the two biomarkers being underexpressed.

Our data indicated that AHF was as sensitive as immunodetection and could be used to simultaneously detect biomarkers in the same tumor section and to reveal the spatial proximity between them. This study showed for the first time the application of fluorescent aptamers in multiplexing imaging experiments to label two biomarkers in human GBM tissues. These results confirmed functional results establishing a cross-talk between integrins and EGFR in several tumors, including gliomas [32,62], and raised the possibility that for EGFR- and integrin α5β1-positive patients, combined therapies based on the dual inhibition of both receptors might be of interest.

## 5. Conclusions

Though the road to using aptamers for the measurement of biomarker expression in tumors is still long, as only a few studies on aptamers have been conducted, our results confirm that aptamers could be alternative molecular probes for histology. Their unique properties would offer advantages in clinics over antibodies, such as shorter reaction time, identical or higher labeling properties, no cross-immunoreactivity issues, and far from being the least, the possibility of easy multiplex analyses, without stripping, of the same section, thus also reducing the need for valuable precious materials such as those from rare donors. We demonstrated the application value of AHF in the detection of integrin α5β1 and EGFR, two biomarkers with wide-ranging cooperation in GBM. We believe that aptamers might have a role to play in multiplexing experiments either using multiple aptamers or through combinations of aptamers/antibodies for the detection of different biomarkers, as alternatives to classical IHC for tumor diagnosis, representing a step towards the multiparameter analysis of whole section tissues.

## Figures and Tables

**Table 1:** Affinity (K_D_) of the interaction between aptamers and cells, determined using flow cytometry.

Aptamer	Target	Glioblastoma Cell Lines	K_D_	Reference
H02	Integrin α5β1	U87MG α5+ (expressing α5 integrin)	277.8 ± 51.8 nM	[44]
E07	EGFR	U87MG EGFR WT (expressing EGFR)	208.7 ± 45.6 nM	Current study

## Data Availability

Data supporting reported results can be provided by the corresponding author upon request.

## Supplementary Materials

The following supporting information can be downloaded at: https://www.mdpi.com/article/10.3390/pharmaceutics14101980/s1, Table S1: Information on aptamers used in this study, Figure S1: Detection of EGFR using IHF and AHF in MCF7 and MDA-MB-231 cells, Figure S2: Light microscopy with H&E dye of a section adjacent to that shown in Figure 4, Figure S3: Surface plot showing the intensity profile of cells represented in Figure 4A.
