# Supplementary material for: Bioimaging Nucleic-Acid Aptamers with Different Specificities in Human Glioblastoma Tissues Highlights Tumoral Heterogeneity
Source: Pharmaceutics. 2022 Sep 20;14(10):1980. doi: 10.3390/pharmaceutics14101980 (PMC9609998; doi:10.3390/pharmaceutics14101980)
Supplement: Supplementary file 1 [file pharmaceutics-14-01980-s001.zip › pharmaceutics-1859154-supplementary.pdf]

Table S1. Information on aptamers used in this study: nucleic acid type, length, sequence, reference of the papers in which their identification was described.

Target	Aptamer name-*conjugated fluorophore*	Type	Length	Aptamer sequence-*conjugated fluorophore*	References
Integrin α5β1	H02-*Cy5*	2’F-RNA	68-mer	5’-GGUUACCAGCCUUCACUGCGGACGGACAGAGAGUGCAACCUGCCGUGCCGCACCACG GUCGGUCACAC-*Cy5*-3’	[44]
EGFR	E07-*Cy5*	2’F-RNA	48-mer	5’-*Cy5*-GGACGGAUUUAAUCGCCGUAGAAAAGCAUGUCAAAGCCGGAACCGUCC-3’	[45, 48]
	E07-*Al488*	2’F-RNA	48-mer	5’-*Al568*-GGACGGAUUUAAUCGCCGUAGAAAAGCAUGUCAAAGCCGGAACCGUCC-3’	

Cy5 stands for Cyanine 5, *Al* stands for Alexa, 2’F stands for 2’-fluoro-modified pyrimidine nucleotides

Figure S1

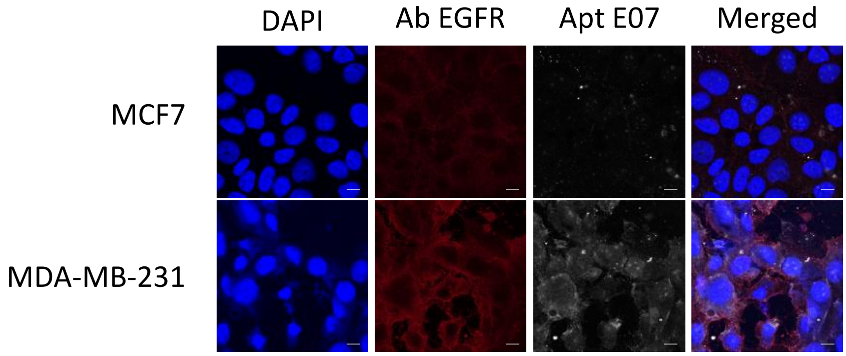

Figure S1. Detection of EGFR by IHF and AHF on cells MCF7 and MDA-MB-231. Cells were seeded in coverslips and incubated with 100 nM of aptamer E07 for 30 minutes. The aptamer, labeled with Cy5, is represented in white. Incubation of antibody anti-EGFR was followed by incubation with a secondary antibody labelled with Alexa 568 (represented in red). Nuclei are stained with DAPI (blue). Scale bar = 10 μm.

Figure S2

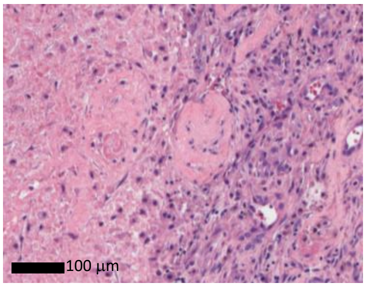

Figure S2. Light microscopy with H&E dyes of a section adjacent to that shown in Figure 4A.

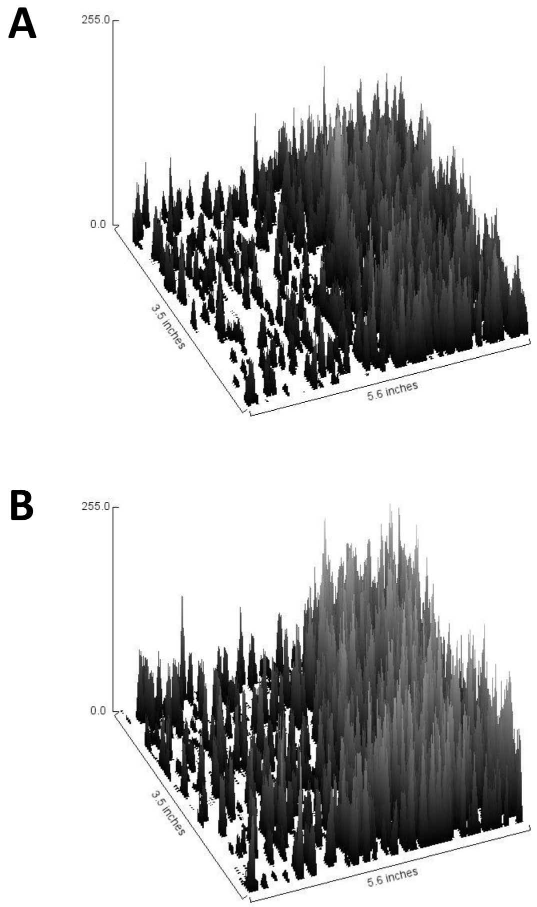

Figure S3. Surface plot showing the intensity profile of cells from Figure 4A which further defines two areas on the left and right of the plot. Briefly, DAPI images from Figure 4A were used. **A**. was obtained from the top DAPI image in Figure 4A and **B.** was obtained from the bottom DAPI image in Figure 4A. Surface plots were defined using ImageJ, by selecting the mean intensity of nuclei staining along a median x-axis with a width of 1000 pixels.
